# Aplicando o EASIX na Osteoartrite. O Elo Entre Inflamação e Risco Cardiovascular

**DOI:** 10.36660/abc.20250314

**Published:** 2025-08-06

**Authors:** Marcio Roberto Moraes de Carvalho

**Affiliations:** 1 Universidade Federal Fluminense Niterói RJ Brasil Universidade Federal Fluminense, Niterói, RJ – Brasil

**Keywords:** Endotélio, Doenças Cardiovasculares, Osteoartrite

A osteoartrite (OA), há muito considerada uma doença articular de desgaste mecânico, está sendo cada vez mais reinterpretada à luz das evidências que a identificam como uma doença inflamatória crônica de baixo grau. Essa mudança é mais do que semântica; ela remodela fundamentalmente a forma como avaliamos os riscos sistêmicos enfrentados por esses pacientes, particularmente o risco cardiovascular (RCV). Dado o crescente reconhecimento da disfunção endotelial como uma manifestação-chave do processo inflamatório envolvido no desenvolvimento de eventos vasculares, a busca por ferramentas capazes de detectar indicadores desses mecanismos subjacentes é de fundamental importância.^1^

Nesta edição,^2^ apresentamos uma nova aplicação do Índice de Ativação e Estresse Endotelial (EASIX) para prever o RCV em pacientes com OA. Originalmente desenvolvido para condições hematológicas, particularmente a doença do enxerto contra o hospedeiro (DECH), o EASIX integra três marcadores amplamente disponíveis – LDH, creatinina sérica e contagem de plaquetas – em um único índice que reflete o estresse endotelial.^3 , 4^

Validado em diversos contextos marcados por disfunção endotelial, incluindo DECH aguda, microangiopatia trombótica associada a transplante, sepse e pancreatite aguda, o valor medido pelo EASIX está associado a maior morbidade e mortalidade.^5 , 6^ Sua nova aplicação oferece uma ferramenta prática e fisiologicamente sólida para o monitoramento de complicações cardiovasculares em pacientes com OA.^6 , 7^

O estudo^2^ aplicou o escore EASIX a uma coorte de pacientes com OA com idade entre 40 e 79 anos, excluindo aqueles com diagnóstico conhecido de doença cardiovascular (DCV). Os resultados demonstraram que um escore EASIX mais alto foi significativamente associado a um risco aumentado de DCV aterosclerótica (OR: 1,94; IC de 95%: 1,57–2,41) e mortalidade por todas as causas (HR: 1,59; IC de 95%: 1,14–2,23). Especificamente, indivíduos com log (EASIX) > −0,78 apresentaram maior risco de morte por qualquer causa em comparação àqueles com log (EASIX) < −1,29. As análises de subgrupos revelaram que essas associações foram particularmente pronunciadas entre pacientes com idade ≥ 65 anos, mulheres e indivíduos negros, destacando potenciais variações demográficas na utilidade prognóstica da pontuação EASIX na população com OA.

O paralelo entre inflamação e regeneração tecidual na OA é complexo, pois envolve fatores locais e sistêmicos.^8^ A disfunção endotelial, central à aterosclerose e às doenças cardiovasculares, pode explicar, em parte, o RCV elevado na OA. A inflamação crônica na OA promove a liberação sistêmica de mediadores inflamatórios, acelerando o dano endotelial e a progressão da aterosclerose.^9^ A inflamação de baixo grau, mediada principalmente pelo sistema imunológico inato, promove a degradação da cartilagem, a remodelação óssea e as alterações sinoviais. Citocinas e quimiocinas não apenas promovem danos estruturais, mas também prejudicam a regeneração tecidual, um papel duplo bem documentado na artrite reumatoide^10^ e na OA.

Este editorial levanta uma questão central: a inflamação é o elo entre doenças degenerativas e adquiridas? Evidências emergentes sugerem que a inflamação crônica de baixo grau está subjacente a algumas condições, como aterosclerose, OA e diabetes tipo 2,^11^ atuando tanto como um impulsionador da degeneração quanto como um gatilho frustrado da regeneração. Reconhecer a inflamação como um vértice fisiológico central abre novas oportunidades terapêuticas e diagnósticas.

Naturalmente, ainda existem limitações. O EASIX ainda não foi validado prospectivamente nesta população. Questões sobre valores de corte específicos, reprodutibilidade interlaboratorial e o impacto das comorbidades precisam ser abordadas. No entanto, essas questões em aberto não diminuem a força do raciocínio fisiopatológico subjacente.

Este editorial vê neste estudo uma semente conceitual fértil, vislumbrando um futuro em que o RCV é avaliado por meio de inflamação, microangiopatia e estresse endotelial, oferecendo novas bases para a cardiologia tradicional. Nesse contexto, o EASIX pode transcender seu papel numérico para preencher a lacuna entre a reumatologia degenerativa e a cardiologia preventiva. A medicina será cada vez mais transversal, inflamatória e endotelial, e o uso do EASIX pode ser uma oportunidade para essa compreensão.
