# Supplementary material for: Aplicando o EASIX na Osteoartrite. O Elo Entre Inflamação e Risco Cardiovascular
Source: Arq Bras Cardiol. 2025 Aug 6;122(7):e20250314. [Article in Portuguese] doi: 10.36660/abc.20250314 (PMC12695101; doi:10.36660/abc.20250314)
Supplement: Translation

# Translation: Applying EASIX in Osteoarthritis. The Link between Inflammation and Cardiovascular Risk

**DOI:** 10.36660/abc.20250314i

**Authors:** Marcio Roberto Moraes de Carvalho

**Affiliations:** 1 Niterói RJ Brazil Universidade Federal Fluminense, Niterói, RJ – Brazil

**Keywords:** Endothelium, Cardiovascular Diseases, Osteoarthritis

Osteoarthritis (OA), long regarded as a mechanical wear-and-tear disease of the joints, is increasingly being reinterpreted in light of evidence identifying it as a chronic low-grade inflammatory disease. This shift is more than semantic; it fundamentally reshapes how we assess the systemic risks faced by these patients, particularly their cardiovascular risk (CVR). Given the growing recognition of endothelial dysfunction as a key manifestation of the inflammatory process involved in vascular event development, the pursuit of tools capable of detecting indicators of these underlying mechanisms is of substantial importance.^1^

In this issue,^2^we present a new application of the Endothelial Activation and Stress Index (EASIX) to predict CVR in OA patients. Originally developed for hematological conditions, particularly graft-versus-host disease (GVHD), EASIX integrates three widely available markers - LDH, serum creatinine, and platelet count - into a single index that reflects endothelial stress.^3 , 4^

Validated in diverse contexts marked by endothelial dysfunction, including acute GVHD, transplant-associated thrombotic microangiopathy, sepsis, and acute pancreatitis, the value measured by EASIX is associated with greater morbidity and mortality.^5 , 6^Its new application offers a practical and physiologically sound tool for monitoring cardiovascular complications in OA patients.^6 , 7^

The study^2^applied the EASIX score to a cohort of patients with OA aged 40 to 79 years, excluding those with a known diagnosis of cardiovascular disease (CVD). The results demonstrated that a higher EASIX score was significantly associated with an increased risk of atherosclerotic CVD (OR: 1.94; 95% CI: 1.57–2.41) and all-cause mortality (HR: 1.59; 95% CI: 1.14–2.23). Specifically, individuals with log (EASIX) > −0.78 exhibited a higher risk of death from any cause compared to those with log (EASIX) < −1.29. Subgroup analyses revealed that these associations were particularly pronounced among patients aged ≥65 years, women, and Black individuals, highlighting potential demographic variations in the prognostic utility of the EASIX score within the OA population.

The parallel between inflammation and tissue regeneration in OA is complex, as it involves local and systemic factors.^8^Endothelial dysfunction, central to atherosclerosis and CVD, may partly explain the elevated CVR in OA. Chronic inflammation in OA promotes the systemic release of inflammatory mediators, accelerating endothelial damage and atherosclerosis progression.^9^Low-grade inflammation, mainly mediated by the innate immune system, drives cartilage degradation, bone remodeling, and synovial changes. Cytokines and chemokines not only promote structural damage but also impair tissue regeneration, a well documented dual role in rheumatoid arthritis^10^and OA.

This editorial raises a main question: Is inflammation the link between degenerative and acquired diseases? Emerging evidence suggests that chronic low-grade inflammation underlies some conditions, such as atherosclerosis, OA, and type 2 diabetes,^11^acting as both a driver of degeneration and a frustrated trigger of regeneration. Recognizing inflammation as a central physiological vertex opens up new therapeutic and diagnostic opportunities.

Naturally, limitations remain. The EASIX has not yet been prospectively validated in this population. Questions about specific cut-off values, interlaboratory reproducibility, and the impact of comorbidities need to be addressed. However, these open questions do not diminish the strength of the underlying physiopathological reasoning.

This editorial sees in this study a fertile conceptual seed, envisioning a future in which CVR is assessed through inflammation, microangiopathy, and endothelial stress, offering new foundations for traditional cardiology. In this context, EASIX can transcend its numerical role to bridge the gap between degenerative rheumatology and preventive cardiology. Medicine will increasingly be cross-sectional, inflammatory, and endothelial, and the use of EASIX could be an opportunity for this understanding.
